# Impaired phagocytosis and oxidative respiratory burst activity in sickle cell anemia leukocytes

**DOI:** 10.1016/j.jtumed.2024.07.010

**Published:** 2024-08-10

**Authors:** David B. Akinbo, Olutayo I. Ajayi, Onyinye M. Eluji, Imisioluwa Olatunji, Temisan M. Okoroloko

**Affiliations:** a Clinical Research Services, Holden Comprehensive Cancer Center, University of Iowa Health Care, Iowa City, IA, USA; b Haematology and Blood Transfusion Science Unit, Department of Medical Laboratory Science, College of Medicine and Health Sciences, Afe Babalola University, Ado – Ekiti, Ekiti, Nigeria; c Department of Physiology, School of Basic Medical Sciences, College of Medical Sciences, University of Benin, Edo State, Nigeria

**Keywords:** فقر الدم المنجلي, خلل كريات الدم البيضاء, الالتهابات البكتيرية, نسبة كريات الدم البيضاء, الوفيات, Bacterial infections, Leukocyte dysfunction, Leukocyte ratio, Mortality, Sickle cell anemia

## Abstract

**Objectives:**

This case-control study investigated the mode of leukocyte function in sickle cell anemia (SCA) to delineate the underlying immunopathology for early diagnosis and mitigate the increased bacterial infection risk in this patient population.

**Method:**

In total, 90 participants comprising 24 hemoglobin (Hb)-AA, 22 Hb-AS, 23 steady state Hb-SS and 21 vaso-occlusive crisis state Hb-SS subjects were recruited for this study. The subjects were further divided into the following six groups: Hb-AA and Hb-AS subjects as control groups, Hb-SS subjects at steady state, Hb-SS subjects in a vaso-occlusive crisis state, Hb-SS subjects undergoing medication (Meds), and Hb-SS subjects undergoing medication plus blood transfusion (Meds/BT) group, respectively. Hematological analysis, Hb electrophoresis, leukocyte ratios, and leukocyte functional assays were assessed with standard methods, and interleukin 8 (IL-8) and L-selectin levels were evaluated using enzyme-linked immunosorbent assays.

**Results:**

Total leukocyte and monocyte counts were increased in the Hb-SS groups compared to the control groups. However, the Hb-SS groups had lower lymphocyte counts than the other groups (p < 0.005). Leukocyte viability was increased in the SCA groups, while phagocytic activities and oxidative respiratory burst were both reduced in the SCA groups (p < 0.005). Increased IL-8 levels were observed in all SCA groups (p < 0.05), whereas L-selectin levels of the Hb-SS steady and Hb-SS on Meds groups were decreased compared to the other groups (p < 0.05). The neutrophil-to-lymphocyte ratio, monocyte-to-lymphocyte ratio, and platelet-to-lymphocyte ratio were higher in the SCA groups than the control groups (p < 0.05).

**Conclusion:**

Impaired leukocyte phagocytic and oxidative respiratory burst activities constitute altered leukocyte function in SCA, which can increase their susceptibility to infections and the risk of mortality, especially during the crisis state. Novel therapeutic approaches can be tailored specifically to enhance these leukocyte functions and mitigate the increased infection risk in SCA.

## الملخص

**أهداف البحث:**

بحثت دراسة الحالات والشواهد هذه في وضع وظائف الكريات البيضاء في فقر الدم المنجلي لتحديد الأمراض المناعية الأساسية للتشخيص المبكر والتخفيف من خطر العدوى البكتيرية المتزايدة في هذه الفئة من المرضى.

**طريقة البحث:**

تسعون مشاركا يضمون 24 هيموجلوبين-أ أ و22 هيموجلوبين-أ س و23 حالة ثابتة من هيموجلوبين-س س و21 حالة أزمة انسداد الأوعية الدموية هيموجلوبين-س س تم استقطابهم لهذه الدراسة. تم تصنيف الموضوعات أيضا إلى ست مجموعات، أي موضوعات هيموجلوبين-أ أ وموضوعات هيموجلوبين-أ س كمجموعات مراقبة، موضوعات هيموجلوبين-س س في حالة مستقرة، موضوعات هيموجلوبين-س س في حالة أزمة انسداد الأوعية الدموية، موضوعات هيموجلوبين-س س الخاضعة للأدوية، ومجموعة هيموجلوبين-س س التي تخضع للعلاج بالإضافة إلى مجموعة نقل الدم على التوالي. تم تقييم تحليل الدم، والرحلان الكهربائي للهيموجلوبين، ونسب الكريات البيضاء، والمقايسات الوظيفية للكريات البيضاء باستخدام الطرق القياسية، وتم تقييم مستويات انترليوكن-8 و إل-سيليكتن باستخدام إليزا.

**النتائج:**

تم زيادة إجمالي عدد الكريات البيضاء والوحيدات في مجموعات هيموجلوبين-س س مقارنة بمجموعات التحكم. ومع ذلك، كان لدى مجموعات هيموجلوبين-س س عدد أقل من الخلايا الليمفاوية مقارنة بالمجموعات الأخرى. تمت زيادة صلاحية كريات الدم البيضاء في مجموعات فقر الدم المنجلي، في حين تم تقليل أنشطة البلعمة والانفجار التنفسي التأكسدي في مجموعات فقر الدم المنجلي. لوحظت زيادة في مستويات انترليوكن-8 في جميع مجموعات فقر الدم المنجلي، ومع ذلك انخفضت مستويات إل-سيليكتن لـ هيموجلوبين-أ س الثابت و هيموجلوبين-أ س في مجموعات الأدوية مقارنة بالمجموعات الأخرى. تم العثور على نسبة العدلات إلى الخلايا اللمفاوية، ونسبة الخلايا الليمفاوية الوحيدة ونسبة الصفائح الدموية إلى الخلايا الليمفاوية لمجموعات فقر الدم المنجلي أعلى من مجموعات التحكم.

**الاستنتاجات:**

يشكل خلل أنشطة البلعمة والانفجار التنفسي التأكسدي لكريات الدم البيضاء في مجموعات مرض فقر الدم المنجلي، مما قد يزيد من قابليتها للإصابة بالعدوى وخطر الوفاة، خاصة أثناء حالة الأزمة. يمكن تصميم الأساليب العلاجية الجديدة خصيصا لتعزيز وظائف كريات الدم البيضاء هذه والتخفيف من خطر الإصابة المتزايد في مرض فقر الدم المنجلي.

## Introduction

Sickle cell disease (SCD) is one of the most prominent genetic diseases worldwide, with a very high prevalence in the Middle East, Mediterranean regions, Southeast Asia, and sub-Saharan Africa. It is characterized by the inheritance of two abnormal hemoglobin (Hb) genes, with one being responsible for the sickle Hb (Hb-S) production. SCD comprises the homozygote Hb-S and compound heterozygotes Hb-S including Hb-SC, SD, SO-Arab, and Sβ-thal. People who inherit one sickle mutation (Hb-S) have the sickle cell trait (Hb AS), an otherwise largely benign condition that confers some protection against severe malaria, which is the reason SCD is more prevalent in parts of the world where malaria is endemic.^1^^,^^2^ More than three-quarters of the more than 500,000 annual births with SCD are found in sub-Saharan Africa, especially Equatorial Guinea, Benin, Burkina Faso, Nigeria, Sierra Leone, and Togo. Sickle cell anemia (SCA) is the most common and severe form of SCD, with about 50–90% of affected individuals experiencing acute vaso-occlusive crises and a reduced life expectancy of about 30 years compared to the general population.^3^^,^^4^ SCA is associated with significant morbidity and mortality due to its unique abnormal red blood cell (RBC) morphology. It causes recurrent vaso-occlusive events, chronic hemolysis, endothelial dysfunction, long-term complications such as avascular necrosis, and impaired leukocyte functions, significantly compromising the immune system and leading to high susceptibility to invasive bacterial infections and severe malaria complications. The frequent predisposition of SCA patients to severe infections is one of the major precipitants of the sickle cell crisis, and the leading cause of morbidity and overall mortality among SCA patients, especially in developing countries.^5^^,^^6^

While much attention has been given to the pathophysiology of RBC abnormalities in SCA, there is a dearth of knowledge on the role of leukocyte dysfunction in the disease phenotype. Specific dysfunctions within the leukocyte population including neutrophils, monocytes, and lymphocytes have been previously reported. These cells play crucial roles in immune surveillance and host defense mechanisms.^7^^,^^8^ Leukocyte dysfunction results in increased susceptibility to infections in SCA. Neutrophils are the first line of defense against invading bacteria, and they exhibit impaired chemotaxis, phagocytosis, and oxidative burst activity in SCA patients, thus compromising the ability of the patient with SCA to effectively eliminate invading pathogens.^9^ SCA monocytes also exhibit altered adhesive properties, cytokine production, and phagocytic activity. Monocytes are considered highly essential mediators of innate immunity, and their dysfunction can further impair the host's defense, heightened inflammatory responses, and overall immune response.^10^^,^^11^ It is therefore imperative to understand the intricate mechanisms of leukocyte dysfunction in SCA to delineate the underlying immunopathology for developing potential targeted interventions to alleviate their increased infection risk.

## Materials and Methods

### Study design and patient selection

This case-control study was conducted between July 2019 and January 2020 among children and young adults aged 10–40 years homozygous for SCA, as well as their relatives and staff in a single reference center in the Southern region of Nigeria (The Sickle Cell Foundation, Benin City, Edo State, Nigeria). Written informed consent was obtained from all participants as well from guardians/parents of subjects below 18 years old. In total, 90 participants comprising 23 steady state Hb-SS patients, 21 vaso-occlusive crises state Hb-SS patients, 22 Hb-AS, and 24 apparently healthy persons with Hb-AA genotype were recruited for this study. The participants were further classified into the following six groups: 23 Hb-SS subjects at steady state, 21 Hb-SS subjects in the vaso-occlusive crisis state, 13 Hb-SS subjects undergoing medication alone (Meds) (15–35 mg/kg/day hydroxyurea, 1–5 mg/d folic acid, malaria prophylaxis, penicillin prophylaxis, and analgesics), 8 Hb-SS subjects undergoing medication and blood transfusion (Meds/BT) (with a minimum of 1 blood transfusion procedure in the 6 months prior to study entry), 22 Hb-AS, and 24 Hb-AA.

Steady state Hb-SS subjects were characterized by the absence of acute complicating factors or clinical symptoms, no vaso-occlusive crisis events, and pain scores ≤3 for at least 3 months. However, Hb-SS subjects with acute exacerbation of symptoms including bone/joint pains or pain in multiple sites/tissue ischemia with pain scores ≥5 and requiring hospitalization at the time of enrollment were clinically considered being in a vaso-occlusive crisis state. Control subjects having routine blood tests without hemoglobinopathy, no major infectious episodes in the 12 months prior to the study, and not on anti-inflammatory drugs during the 2 weeks preceding the study were frequency matched to SCD patients by age, sex, and ethnicity for the study. SCD severity was assessed by a careful history, complete physical examination, and using an objective score in a modification of the Akinlade and colleagues’ structured outcome measures.^12^ All subjects with a history of established endocrine dysfunctions, concurrent autoimmune disorders or other hematological conditions, chronic diseases, or infections including human immunodeficiency virus or hepatitis were excluded from the study. Subjects who were pregnant or lactating subjects at the time of enrollment were also excluded.

### Sample preparation

Venous blood (5 mL) was obtained from each subject and 2.5 mL of each was dispensed into dipotassium ethylene diamine tetra-acetic acid (K_2_EDTA) and lithium heparin tubes, respectively, and gently mixed. Both tubes were labeled appropriately after collection, were always protected from light using sheets of black plastic, and placed in a cool box containing ice packs. The K_2_EDTA blood was used for Hb genotype confirmation via Hb electrophoresis on cellulose acetate strips, and determination of white blood cell and differential count, platelet count, and indices were all performed within 2 h of sample collection. The lithium heparin samples were stored at a temperature of 2–8 °C, and thereafter used to assess leukocyte function and other biochemical analyses.

### Hematological analysis

Complete blood counts of the various participants were analyzed using the automated Sysmex Hematology Analyzer (KX-21N, Osaka, Japan) according to the manufacturer's instructions. The leukocyte subsets were counted after staining peripheral blood smears with Leishman's stain, while the Hb genotype electrophoretic pattern was determined using alkaline Hb electrophoresis as previously described.^13^

### Leukocyte function assay

Circulating leukocytes were isolated from the heparinized whole blood by density gradient centrifugation over an equal volume of Ficoll–Paque (Histopaque®) at densities of 1.077 and 1.119 g/mL within 3 h of collection.^14^ Thereafter, aliquots were dispensed into vials properly labeled with subject identifiers, and stored at 2–8 °C to maintain cell viability before analysis within 24 h of collection. The granulocyte layer was collected and washed in phosphate-buffered saline after hemolysis of unwanted cells. Leukocyte viability was assessed using the trypan blue dye exclusion test, involving incubation of 100 μL cell suspensions and 0.4% trypan blue dye for 5 min in the dark at room temperature. The cells were counted using a counting chamber, and the percentage of unstained cells was calculated as the viable cells.^15^

The leukocyte chemotaxis was determined using the Boyden Chamber with a modified double membrane (Sigma–Aldrich, St. Louis, MO, USA). Cell suspensions (100 μL) were dispensed into the membrane chamber and incubated with 150 μL N-formyl-met-leu (Sigma–Aldrich) as chemoattractant added to the wells of the feeder tray for 6 h. Two membranes were used together to separate the chamber wells: a 5 μm pore size polycarbonate membrane, 22 μm in thickness to select the cells, a 8 μm pore size cellulose membrane of 160 μm in thickness directly above the lower well to trap the cells. Cells from the top side of the membrane chamber were aspirated and placed into 150 μL pre-warmed cell detachment solution pipetted into the cell harvesting tray, prior to the end of incubation. Then 50 μL of 4X Lysis Buffer and Cyrant Quant® GR dye solution were added to each well, incubated for 20 min at room temperature, and read with a fluorescence plate reader at 520 nm.^16^

The Phagocytosis Assay/Phagotest was performed using standard methods. Briefly, 100 μL cell suspension and 10 μL of 1 × 10^7^ *Escherichia coli*/mL were incubated together for 20 min and 90 min at 37 °C, respectively, in a 10% CO_2_ incubator for phagocytosis/killing or growth of the bacteria. Extracellular organisms were removed by washing, and the number of surviving bacteria was determined pre- and post-incubation by measuring their ability to reduce the yellow tetrazolium salt, 3-(4,5-dimethylthiazol-2-yl) 2,5-diphenyltetrazolium bromide (MTT) (Sigma–Aldrich) to a purple formazan product using a microtiter plate reader at 570 nm.^17^ The oxidative respiratory burst activity and degranulation of granulocytes were assessed by the reduction of nitroblue tetrazolium (NBT) dye in the presence of reactive oxygen species. Within 1 h of specimen collection, 100 μL heparinized blood was mixed with an equal amount of 0.2% NBT solution (Sigma–Aldrich) and 25 μL E*. coli* endotoxin, and incubated at 37 °C for 15 min. The resulting solution was gently mixed, and blood smears were made and stained with Leishman's stain. The percentage of cells containing reduced dark-blue formazan was scored microscopically using oil immersion objectives. Cells with large blue black deposits were classified as NBT-positive neutrophils.^18^

### Interleukin 8 and L-selectin quantification

Heparinized blood from each patient was centrifuged within 3 h of collection, and their plasma was stored in aliquots at −80 °C until analysis. Circulating interleukin 8 (IL-8) and soluble L-selectin were assayed in duplicate using commercial enzyme-linked immunosorbent assay kits (Hangzhou East Biopharm, Hangzghou, China) according to the manufacturer's instructions.

### Statistical analysis

Data collected were analyzed using IBM Statistical Product and Service Solutions (SPSS) version 25.0 for Windows (Armonk, NY, USA) and GraphPad Prism version 8.0 for Windows (La Jolla, CA, USA). The results are expressed as the mean ± standard error of the mean (SEM) for each group. Analysis of variance was carried out to test for uniformity at p < 0.05 among the groups. Duncan's multiple range test was used to separate the heterogeneous groups and test the significance of association between categorical variables.

## Results

The study population comprised 56 (62.2%) females and 34 (37.8%) males with a mean age of 20.25 ± 1.81 for Hb-AA, 22 ± 1.62 for Hb-AS, 19.87 ± 1.30 for steady state Hb-SS, and 17.43 ± 1.09 for vaso-occlusive crisis state Hb-SS subjects. Table 1 shows the leukocyte count and subpopulations, platelet count, and leukocyte chemotactic activity of the study participants. There was an increase in total white blood cell count for all Hb-SS (SCA) groups compared to control groups (p < 0.005). The Hb-SS on Meds/BT group had significantly higher lymphocyte count compared to the Hb-SS steady and Hb-SS on Meds groups (p < 0.005). The SCA groups displayed an increased monocyte count (p < 0.05). There were increased platelets in the Hb-SS crisis and Hb-SS on Meds groups compared to the other groups (p < 0.05). No significant differences were observed in neutrophil count and leukocyte chemotactic activity in the study groups (p > 0.05).

**Table 1:** Leukocyte count and subpopulations.

Parameter	Hb-AA (n = 24)	Hb-AS (n = 22)	Hb-SS steady (n = 23)	Hb-SS crisis (n = 21)	Hb-SS on Meds (n = 13)	Hb-SS on Meds/BT (n = 8)	P
TWBC (×10^3^/μL)	5.08 ± 0.18^a^	4.57 ± 1.32^a^	13.27 ± 2.51^b^	12.61 ± 2.09^b^	11.45 ± 1.72^b^	8.97 ± 1.44^b^	<0.001
LYM (%)	42.13 ± 0.97^ab^	40.00 ± 1.87^ab^	35.50 ± 3.37^b^	37.50 ± 3.21^ab^	33.41 ± 2.66^b^	44.75 ± 2.48^a^	0.005
MON (%)	5.60 ± 0.39^b^	5.33 ± 0.51^b^	10.13 ± 0.99^a^	9.93 ± 0.75^a^	9.82 ± 0.80^a^	10.63 ± 0.82^a^	<0.001
Platelets (×10^3^/μL)	205.42 ± 8.2^b^	202.58 ± 17^b^	307 ± 27.83^b^	507.29 ± 74.19^a^	420.18 ± 52.27^a^	346.25 ± 58.64^b^	<0.001
NEUT (%)	52.27 ± 0.93	54.67 ± 1.94	53.38 ± 3.75	52.57 ± 3.29	56.77 ± 2.95	44.63 ± 3.10	0.125
EOS (%)	0.2 ± 0.02	0.2 ± 0.01	0.4 ± 0.02	0.3 ± 0.01	0.3 ± 0.04	0.2 ± 0.00	0.160
BASO (%)	0.06 ± 0.01	0.08 ± 0.02	0.1 ± 0.02	0.2 ± 0.04	0.1 ± 0.06	0.08 ± 0.02	0.023
CHM (RFU)	45.85 ± 1.95	43.78 ± 2.92	47.24 ± 2.57	47.00 ± 2.64	46.58 ± 2.12	48.63 ± 3.67	0.575

BASO: Basophils; CHM: Chemotactic activity; EOS: Eosinophils; LYM: Lymphocytes; MON: Monocytes; NEUT: Neutrophils; TWBC: total white blood cell.

Different letters represent significant differences at p < 0.05.

### Leukocyte functions of the study population

**Figure 1:**
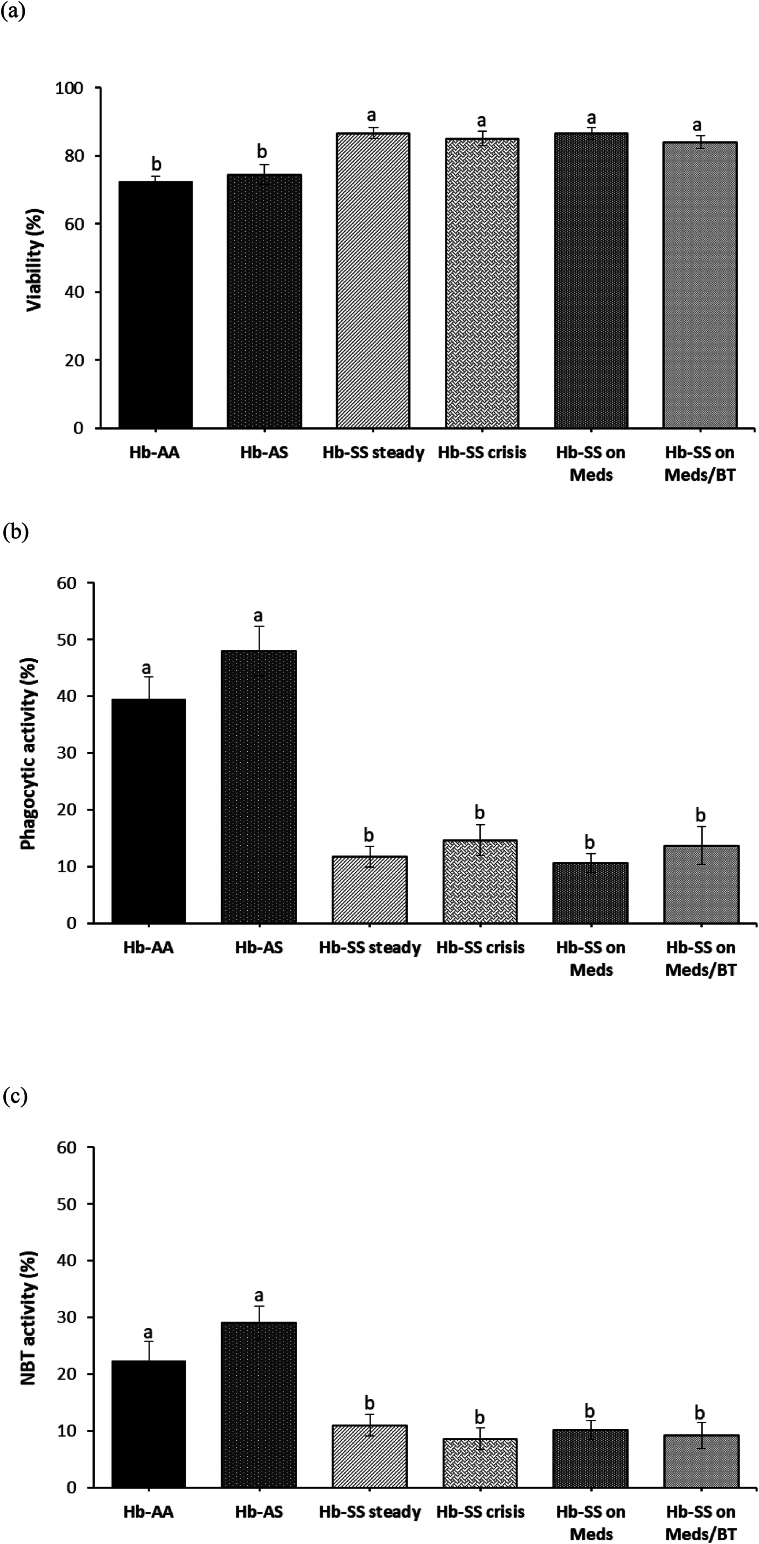
(a–c) Leukocyte functions of the SCA and control groups (a) Percentage of leukocyte viability (b) Percentage of phagocytic activity (c) Percentage of oxidative respiratory burst - NBT. Bars represent the mean ± SEM (n = 90). Bars with different letters are significantly different at p < 0.05.

Cell viability was higher in all SCA groups than in both control groups (p < 0.05; Figure 1a). The SCA groups had reduced phagocytic activity compared with the control groups (p < 0.05; Figure 1b). SCA groups displayed decreased oxidative respiratory burst (NBT) activities compared with the control groups (p < 0.05; Figure 1c). The total white blood cell count (TWBC) negatively correlated with phagocytic activity (PHA) in the Hb-SS crisis group (r = −0.45, p = 0.043). However, no significant correlation was observed between leukocyte count and phagocytic activity, leukocyte subpopulations, platelets, and other leukocyte functions of the other SCA groups and control groups ((p > 0.05; data not shown).

### Plasma levels of IL-8 and L-Selectin

**Figure 2:**
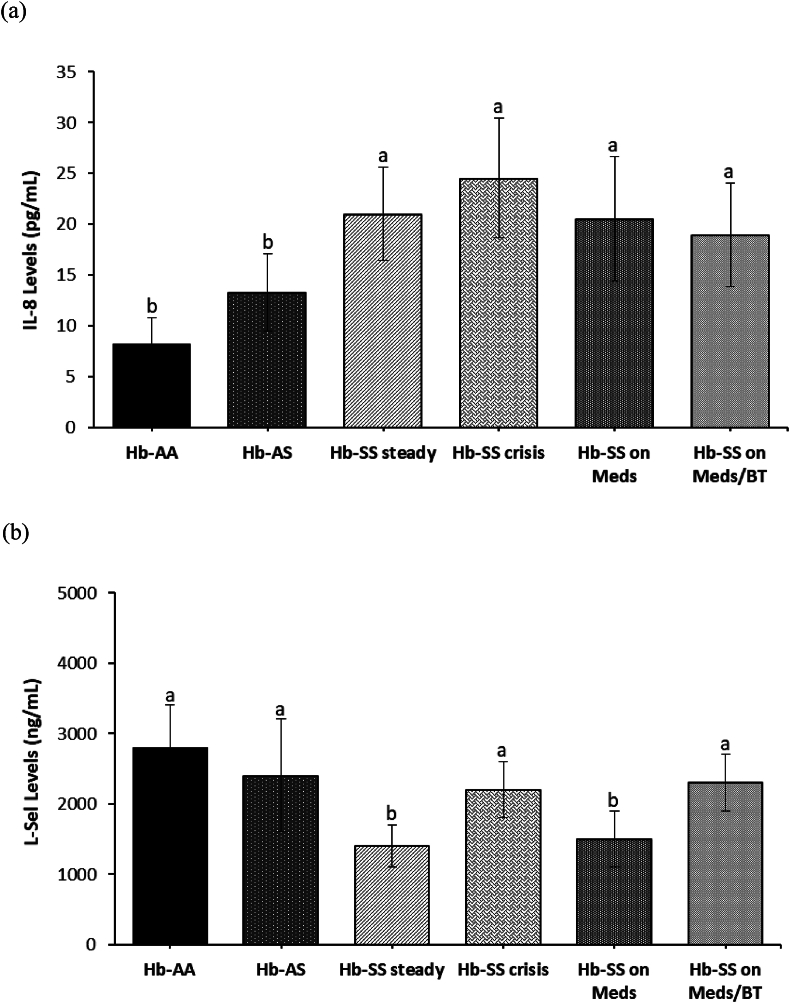
(a–b) Plasma IL-8 and L-Selectin levels (a) IL-8: interleukin 8 levels (b) L-Sel: L-selectin levels. Bars represent the mean ± SEM (n = 90). Bars with different letters are significantly different at p < 0.05.

Increased levels of IL-8 were found in all SCA groups compared with the Hb-AA and Hb-AS groups (p < 0.05; Figure 2a). The Hb-SS steady and Hb-SS on Meds groups showed a decrease in plasma L-selectin levels compared to the other SCA groups and control groups (p < 0.05; Figure 2b).

### Markers of leukocyte activation and systemic inflammation

**Figure 3:**
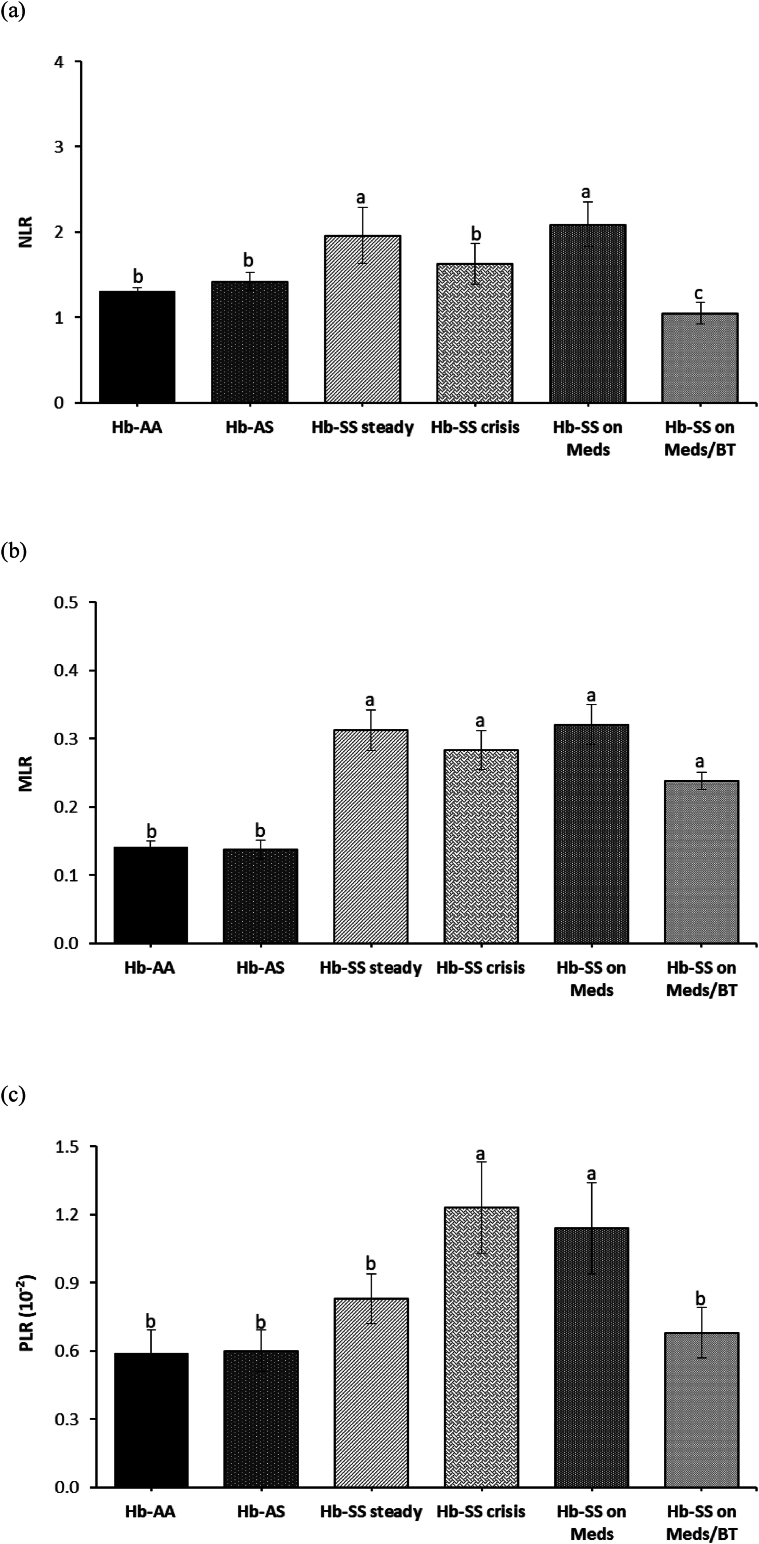
(a–c) Markers of leukocyte activation and systemic inflammation in the SCA and control groups (a) NLR: Neutrophil-to-lymphocyte ratio, (b) MLR: Monocyte-to-lymphocyte ratio (c) PLR: Platelet-to-lymphocyte ratio. Bars represent the mean ± SEM (n = 90). Bars with different letters are significantly different at p < 0.05.

The Hb-SS steady and Hb-SS on Meds groups showed a higher neutrophil-to-lymphocyte ratio (NLR) than the control Hb-AA and -AS groups (p < 0.05). However, the Hb-SS on Meds/BT group displayed a lower NLR than all of the other SCA groups (p < 0.05) (Figure 3a). The monocyte-to-lymphocyte ratio (MLR) was higher in the SCA groups than the control groups (p < 0.05; Figure 3b). A higher platelet-to-lymphocyte ratio (PLR) was observed in the Hb-SS crisis and Hb-SS on Meds groups than in the other SCA groups and control groups (p < 0.05; Figure 3c).

## Discussion

SCA is capable of significantly reducing the life expectancy of millions worldwide; it is a risk factor for bacteremia and overwhelming sepsis.^19^ Leukocyte activation, adhesion, and functions in SCA were assessed by evaluating some hematological parameters, leukocyte function assays, circulating levels of proinflammatory, and a leukocyte adhesion molecule in SCA subjects. The observed negative correlation of total leukocyte count and phagocytic activity in vaso-occlusive crisis SCA patients could further enhance the risk of infection in this population. Bacterial infection associated with leukocytosis has been linked to clinical severity in SCA.^20^^,^^21^ Lymphocytes are integral components of the immune system, mediating the adaptive or protective aspect of inflammatory responses in the body.^22^ The reduced lymphocyte count of the SCA subjects in the study may also account for the susceptibility to infection-mediated acute crises, splenic sequestration, and immune deficiency. This agrees with previous findings of reduced differentiation and circulation of mature lymphocytes in SCA patients, resulting in inadequate memory B-cell function and a diminished humoral immune response.^6^ However, combined treatment involving medications and blood transfusion significantly improved the lymphocyte counts of some of these SCA patients.

The SCA groups displayed higher leukocyte viability among the study groups, suggestive of intact leukocyte membrane integrity in the SCA groups. There was, however, a reduction in the phagocytic activity of the SCA group, in line with previous reports where they associated SCA with decreased phagocytic activity and subsequent susceptibility to infections despite their leukocytosis.^23^ Reduced phagocytic ability of the polymorphonuclear leukocytes may have resulted from the presence of circulating inhibitory factors in the SCA subjects. Therefore, this reduction of phagocytic activity in the SCA groups might represent one of the major mechanisms resulting in the increased susceptibility to overt infections. The highlight of pathogen destruction by phagocytes is the formation of phagosomes, and subsequent release of reactive oxygen species and hydrolytic enzymes into them. However, this will only occur if oxidative respiratory burst activity is adequate.^24^ In this study, the SCA groups showed pronounced decrease in NBT activities, demonstrating impairment in their leukocyte oxidative respiratory burst activity, and is not confounded by medications or blood transfusion. This is consistent with previous findings reporting depressed leukocyte oxidative respiratory burst activity in SCA children and adults.^25^ The susceptibility to infection and impairment of neutrophil oxidative burst activity in SCA is thought to result from hemolysis-derived heme, which causes the induction of heme oxygenase-1 during granulopoiesis, subsequently resulting in the production of neutrophils with reduced oxidative burst capacity.^9^ There was, however, no difference in the chemotactic activity of all groups.

A critical step in bacterial destruction and inflammation is leukocyte extravasation, involving the modulation of adhesion molecules expression on both leukocytes and their counterparts on endothelial cells. The proinflammatory mediator-induced shedding of L-selectin (cluster of differentiation 62 ligand [CD62L]) and elevated CD11b/CD18 expression by the leukocytes are particularly major events in transendothelial migration. L-selectin is an adhesion molecule expressed on the surface of leukocytes, facilitating their adherence to the endothelium and subsequent migration to sites of infection or inflammation.^26^ Hb-SS steady and Hb-SS undergoing medication groups had lower L-selectin levels than the other groups. A decrease in L-selectin levels has been implicated in the shedding process that occurs during neutrophil activation, which can reduce neutrophil's marginal pool and contribute to hyperleukocytosis in the SCA patients.^27^^,^^28^ These impairments may immobilize polymorphonuclear cells on the endothelium, reducing blood flow and promoting microvascular occlusion and vascular damage. Decreased L-selectin levels may also indicate sequestration by binding to activated endothelium.^26^ IL-8 has been used as a functional marker of leukocyte chemotaxis and adhesion, especially in the early catabolic phase.^29^ Elevated IL-8 levels observed in the SCA groups could suggest increased leukocyte chemotaxis and adhesion to the vascular endothelium, and confirm its significant contribution, not only to pathogenesis but also to the chronic inflammatory state in SCA.

Leukocyte count and leukocyte viability were negatively correlated with phagocytic activity in the SCA vaso-occlusive crisis group. In SCA, leukocytosis is usually accompanied by increased susceptibility to infection-mediated acute crises, as evident in the SCA crisis group described above.^20^ The negative association of leukocytosis and leukocyte viability with phagocytosis in the SCA crisis patients indicates that prevalent leukocytosis did not improve their phagocytic activity. This agrees with previous reports on the effect of leukocyte count on some leukocyte functions.^23^ There was, however, no correlation between the leukocyte viability, chemotactic activity; and oxidative respiratory burst activity of the SCA groups, indicating an inappropriate polymorphonuclear cell response. Leukocyte functions can be incorporated as a prognostic tool for identifying patients at higher risk of complications and can facilitate personalized management approaches of SCA patients. Incorporating leukocyte function assays into routine clinical care can also enhance diagnostic accuracy and guide treatment decisions. This can lead to more effective strategies for preventing infections and managing complications in SCA patients, ultimately improving their quality of life and prognosis.

Leukocyte ratios correlate with mortality in the general population and clinical outcomes when elevated in several specific subsets of diseases and chronic disorders including sepsis, pneumonia, COVID-19, and cancer.^30^^,^^31^ There is a keen interest in using leukocyte ratios to evaluate inflammatory status in SCA, given their relative stability against various physiological, pathological, and physical influences. The leukocyte ratios were all increased in the SCA groups, buttressing the contributions of neutrophils, monocytes, lymphocytes, and platelets in the constitutive inflammatory process of SCA.^32^^,^^33^ However, the MLR in this study showed more sensitivity in indicating the ongoing inflammatory process across SCA groups than the other ratios. This correlates with other reports on elevated leukocyte ratios in the SCA patients.^34^ These ratios can thus be used as cost-efficient, stable, and readily available surrogate markers of inflammation. They are a promising marker in the risk stratification of SCA patients and complement established biomarkers in their diagnosis and treatment. Combination treatment with medications and blood transfusion significantly reduced the NLR and PLR of the SCA patients compared to the other SCA groups, suggesting that combination therapy reverses some of the chronic inflammation associated with SCA patients.

This study had certain limitations. Some of our leukocyte function assays may provide limited characterization of the leukocyte subsets, and reduced sensitivity in detecting subtle changes in leukocyte function relative to other sophisticated methods for assessing leukocyte function. Another limitation is that leukocyte ratios as surrogate markers of inflammation may not capture the complex and multifaceted nature of the immune response as well as immune dysregulation. Also, the study had a relatively small sample size with limited age group, which may limit the generalizability of our findings.

## Conclusion

The results of this study suggest that altered modulation of inflammatory markers and leukocyte adhesion molecule could influence leukocyte migration, causing inappropriate leukocytes activation and tissue injury. Reduced leukocyte phagocytic and oxidative respiratory burst activities constitute altered leukocyte function, and may be culpable for the increased susceptibility to infections in SCA and the risk of mortality, especially during the crisis state. Thus, emphasizing their usefulness in predicting poor outcomes in SCA. The leukocyte function in SCA may be improved via promoting anti-inflammatory pathways to mitigate chronic inflammation associated with SCD, which may enhance their phagocytic function. Optimizing the redox balance within leukocytes or modulating oxidative stress pathways could also enhance the oxidative respiratory burst activity of the SCA leukocytes. Future studies investigating the underlying molecular and cellular pathways driving the functional alterations in SCA leukocyte could provide deeper insights into the pathophysiology of their associated immune dysfunction, and help identify novel therapeutic targets and interventions to mitigate their increased infection risk.

## Source of funding

This research did not receive any specific grant from funding agencies in the public, commercial, or not-for-profit sectors.

## Conflict of interest

The authors have no conflict of interest to declare.

## Ethical approval

Ethical approval was granted by the Ethics Committee, Ministry of Health, Edo State. HM.1208/177; 08/14/2017.

## Authors contributions

DBA: Conceptualization, Methodology, Supervision, Validation, Writing – original draft, Writing – review & editing, Funding and resources acquisition. OIA: Conceptualization, Methodology, Supervision, Writing – review & editing of final draft. OME: Investigation, Project administration, Funding and resources acquisition. IO: Data curation, Project administration, Funding and resources acquisition. TMO: Formal analysis, Logistic support, Funding and resources acquisition. All authors have read and agreed to the published version of the manuscript. All authors have critically reviewed and approved the final draft and are responsible for the content and similarity index of the manuscript.
